# Rapid photonic curing effects of xenon flash lamp on ITO–Ag–ITO multilayer electrodes for high throughput transparent electronics

**DOI:** 10.1038/s41598-023-27942-4

**Published:** 2023-01-19

**Authors:** Zhenqian Zhao, Alex Rose, Sang Jik Kwon, Yongmin Jeon, Eou-Sik Cho

**Affiliations:** 1 Department of Electronics Engineering, Gachon University, Seongnam, 13120 Republic of Korea; 2 Department of Biomedical Engineering, Gachon University, Seongnam, 13120 Republic of Korea; 3 PulseForge Corporation, Seoul, 04070 Republic of Korea

**Keywords:** Electrical and electronic engineering, Nanoscience and technology

## Abstract

High-throughput transparent and flexible electronics are essential technologies for next-generation displays, semiconductors, and wearable bio-medical applications. However, to manufacture a high-quality transparent and flexible electrode, conventional annealing processes generally require 5 min or more at a high temperature condition of 300 °C or higher. This high thermal budget condition is not only difficult to apply to general polymer-based flexible substrates, but also results in low-throughput. Here, we report a high-quality transparent electrode produced with an extremely low thermal budget using Xe-flash lamp rapid photonic curing. Photonic curing is an extremely short time (~ μs) process, making it possible to induce an annealing effect of over 800 °C. The photonic curing effect was optimized by selecting the appropriate power density, the irradiation energy of the Xe-flash lamp, and Ag layer thickness. Rapid photonic curing produced an ITO–Ag–ITO electrode with a low sheet resistance of 6.5 ohm/sq, with a high luminous transmittance of 92.34%. The low thermal budget characteristics of the rapid photonic curing technology make it suitable for high-quality transparent electronics and high-throughput processes such as roll-to-roll.

## Introduction

As demand for electronics that can be attached, worn or implanted on the human body has grown, numerous studies have investigated various form-factors of flexible electronics, and transparent electronics for various applications^1–5^. Transparent and flexible electronics technologies are considered key elements for next generation displays, secondary batteries, solar cells, semiconductors, and healthcare devices^6–9^.

Among the various fundamental technologies needed to achieve transparent and flexible electronics, transparent and flexible electrodes are particularly important. Research on transparent flexible electrodes is being conducted using various materials and processes, including transparent conductive oxides (TCO), 2D materials (MXene, Graphene), conductive polymers, and silver nanowires, with the goal of achieving high transmittance and high conductivity at the same time^10,11^. However, most of these transparent flexible electrode candidates have had difficulty achieving both high transmittance and high conductivity at the same time, due to process complexity, and optoelectrical trade-offs.

Among various electrodes, indium tin oxide (ITO) is the most commonly used transparent electrode because it has excellent characteristics, including both high transmittance and low resistivity (6.68 × 10^–4^ Ω cm)^12–14^. ITO is also used as an anode for various organic electronics including organic light-emitting diodes and polymer solar cells because of its high workfunction characteristics^15,16^. However, because the conductivity of TCOs is typically lower than that of metal-based electrodes, it’s often necessary to lower ITO resistivity to improve the efficiency and power consumption of TCO-based electronic devices. In addition, an ITO electrode also has lower flexibility than a metal-based electrode. This has led to growing interest in the oxide-metal-oxide (OMO) structure, which has both mechanical flexibility and high conductivity. The metal thickness determines the transmittance and contributes to the conductivity of the OMO structure^17–19^. ITO–Ag–ITO multilayer electrodes are widely used because it offers better optical and electrical characteristics than ITO alone^15^. In the OMO structure, the choice of oxide materials is very wide, and can depend on the application^20–26^. However, ITO is a representative oxide candidate that satisfies all conditions, including high work-function, high transmittance and high conductivity, so that it can be used as an anode in organic electronics (organic light-emitting diode, polymer solar cell etc.), which are commonly used in flexible and transparent electronics^15^.

To achieve flexibility, the OMO electrode must be fabricated using a low-temperature process (< 150 °C), so that the flexible polymer substrate is not damaged. However, at low-temperature the non-stoichiometric ratio of deposited ITO films is not balanced^27,28^, and the interface effect between layers will restrict its characteristics. As a result, the ITO–Ag–ITO multilayer must be annealed to improve its characteristics. The crystallinity of amorphous phase ITO–Ag–ITO can be improved by the annealing process. However, commonly used methods such as rapid thermal annealing are difficult to apply in high-throughput processes, because the high thermal budget process exposes the polymer to high temperature (over 300 °C) for a long time (more than 5 min)^29–35^.

This long post-annealing process limits throughput, because it is difficult to use with a roll-to-roll (R2R) process, which is one of the advantages of transparent flexible electronics. Also, the poor thermal stability of Ag film makes it unsuitable for traditional annealing processes^36^. To realize a superior transparent flexible ITO–Ag–ITO electrode with high transmittance and high conductivity, while ensuring high-throughput, a post-annealing process with a low thermal budget is required to provide high transmittance and high conductivity, while ensuring high-throughput. Digital thermal processing using Xenon flash lamp annealing (Xe-FLA) is an ideal curing method that can be applied to heat-sensitive substrates while retaining the effects of previous heat treatments on thin films^37–40^. Many previous reports have examined the digital thermal processing effects on ITO and amorphous silicon thin films during Xe-FLA irradiation^37^.

In this study, we report a high-throughput, high-performance transparent electrode with high transmittance and high conductivity, fabricated with an extremely low thermal budget through rapid photonic curing, based on Xe-flash lamp annealing (Xe-FLA). The Xe-FLA conditions were optimized by simulations, to ensure it could provide a sufficiently high photonic curing effect even in an extremely short time, on the μs-scale, at a high temperature of 800 °C or higher. By optimizing the Xe-FLA irradiation time, radiant power, energy density, and the thickness of the ITO–Ag–ITO electrode, the figure of merit (FOM), which indicates the transmittance and conductivity characteristics, was improved by up to 58%. Using this photonic curing effect, an ITO–Ag–ITO transparent electrode with a high transmittance of 92.34% and a low sheet resistance of 6.5 ohm/sq was demonstrated.

## Results and discussion

### Rapid photonic curing simulation and ITO–Ag–ITO electrode design

To fabricate flexible and transparent electronics using a high throughput roll-to-roll process, it is essential to have a low thermal budget annealing process at a low temperature and a short annealing time, to avoid damage to the polymer-based flexible substrate. Xe-FLA is a high efficiency process which allows short photonic curing in the us time scale, and can anneal the material at a low thermal budget, without damaging the flexible substrate^41–43^. Xe-FLA is one of the thermal annealing methods that can heat thin films in a very short time without damaging the substrate. Various studies have been reported on Xe-FLA based on the heat treatment effect^38–40,44^.

**Figure 1:**
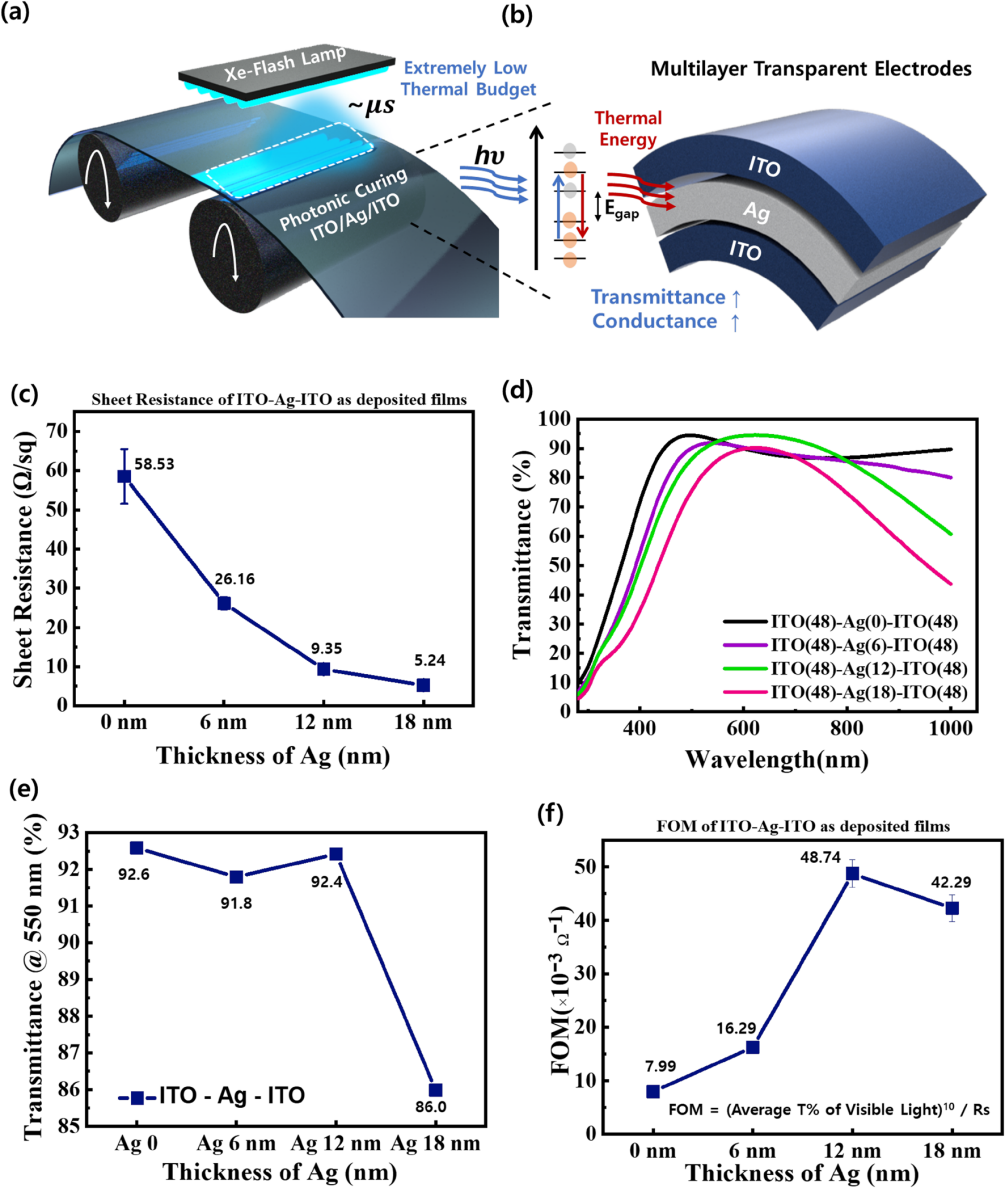
Concept of Rapid Photonic Curing using Xe-FLA and the Electro-optical Design of the ITO–Ag–ITO: (**a**) Schematic illustration of the R2R photonic curing of ITO–Ag–ITO (**b**) Schematic illustration of the ITO–Ag–ITO Xe-FLA mechanism. (**c**) Sheet resistance according to the Ag layer thickness in the multilayer electrode. (**d**) Transmittance according to Ag thickness in the ITO–Ag–ITO electrode. (**e**) Transmittance at 550 nm according to Ag thickness in the multilayer electrode. (**f**) FOM value according to Ag thickness in the multilayer electrode.

Using rapid photonic curing with Xe-FLA, high-quality transparent flexible ITO–Ag–ITO electrodes can be fabricated with high throughput, as shown in Fig. 1a. The transparent electrode undergoes an electron transition after receiving the radiant energy, which in turn releases part of the heat energy from the high energy level to the low energy level, intensifying atomic vibration, causing the material to undergo a phase transformation^45,46^. A rapid photonic curing effect is induced by this mechanism, resulting in simultaneous high transmittance and high conductivity in the ITO–Ag–ITO multilayer. (Fig. 1b).

In this experiment, we used PulseForge 1300 equipment (*PulseForge Corporation*), which uses a xenon light source, and has a full range output spectrum (200–1500 nm), with high radiant energy density (maximum: 45 J/cm^2^) and radiant power density (maximum: 35 kW/cm^2^), which can address a diversity of experimental conditions^44,47^. The Xe-FLA based photonic curing condition is described in more detail in the “Materials and methods” Section.

Before the radiant photonic curing effect of the Xe-FLA was applied, ITO–Ag–ITO electrodes were designed and optimized to have high transmittance and conductance. The thickness of the ITO electrode was fixed at 48 nm, based on the number of sputter scans 0, 1, 2 and 3, and the Ag thickness was approximately 0, 6, 12 and 18 nm, respectively. Since Ag has higher conductivity than ITO, as the thickness of the Ag electrode increased, the ITO–Ag–ITO sheet resistance improved, but the transmittance showed a trade-off characteristic (Fig. 1c, d). When the Ag thickness was 0, 6, 12 and 18 nm, the sheet resistance of the multilayer was 58.53, 26.16, 9.35 and 5.24 ohm/sq, and the average transmittance was 86.2, 82.2, 82.4 and 73.3%, respectively (Fig. S1).

However, adding Ag can improve transmittance in some wavelength ranges. When the Ag thickness was 12 nm, the transmittance was obviously higher in the 557–787 nm range, and the maximum transmittance value was 94.5%. As a result, the transmittance of ITO–Ag–ITO can be influenced by the morphology of the Ag film^31^. Transmittance is also affected by the large amount of valence electrons on the metal surface, since surface plasmon elements will form when the light is incident. This will result in the formation of an electric field stronger than the excited electric field^48^. As a result, the transmittance at 550 nm, the wavelength with the greatest luminance to the human eye, reached a high of 92.4% when the thickness of the Ag was 12 nm (Fig. 1e).

FOM is the ratio of the average transmittance of visible light to sheet resistance, which is equivalent to FOM = $\frac{{\mathrm{T}}_{\mathrm{a}}^{10}}{{\mathrm{R}}_{\mathrm{s}}}$ (T_a_: average transmittance, R_s_: Sheet resistance). Accordingly, a larger FOM value indicates that the optoelectrical characteristics of the ITO–Ag–ITO are relatively better. The FOM of a multilayer with Ag thickness increases is 7.99, 16.29, 48.74 and 42.29 [× 10^–3^ Ω^−1^], respectively (Fig. 1f). It was confirmed that the FOM value of the OMO electrode with the Ag layer was higher than that without an Ag layer. The largest FOM was observed when the Ag layer was 12 nm. These results confirmed that the OMO structure is better than that of an ITO layer. Obviously, when the thickness of the Ag layer is 12 nm, there is a better trade-off between the transmittance and sheet resistance characteristics of the multilayer.

**Figure 2:**
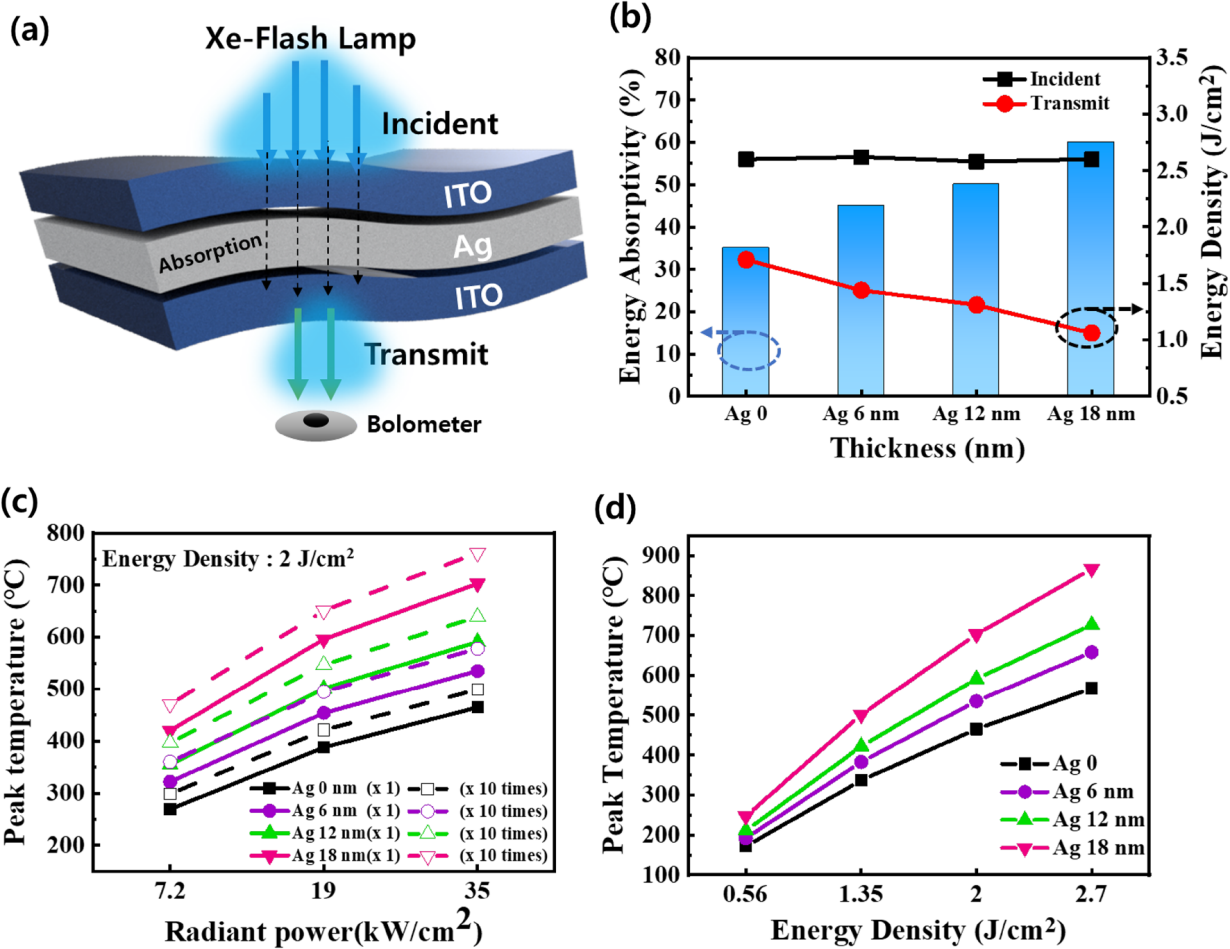
Analysis of Rapid Photonic Curing Annealing Temperature according to the Xe-FLA conditions: (**a**) Schematic illustration of the measured energy of Xe-FLA delivered to the ITO–Ag–ITO electrode. (**b**) Radiant energy absorptivity of different Ag thicknesses in the ITO–Ag–ITO electrode (@ 2.7 J/cm^2^, 37 kW/cm^2^, 100 μs). (**c**) Peak temperature at different Ag thicknesses, number of Xe-FLA irradiations, and radiant powers (@ 2.0 J/cm^2^). (**d**) Peak temperature for different Ag thicknesses and radiant energy density of the ITO–Ag–ITO electrode.

Before rapid photonic curing is applied, the temperature change in the Ag in the multilayer was analyzed considering the absorption of radiant energy, for various Ag layer thicknesses (Fig. 2a). The energy transmission was measured using the bolometer of the PulseForge 1300 device. As shown in Fig. 2b, as the Ag thickness increased, the transmitted energy density decreased, and the energy absorptivity increased. These results suggest that the thicker the Ag layer becomes, the higher the heat temperature that will be transferred to the multilayer.

As shown in Fig. 2c and d, the ITO–Ag–ITO electrode peak temperature changes after absorbing radiant energy, as analyzed by SimPulse (PulseForge Corporation) software simulation. The simulation results in Fig. 2c show that an annealing effect of several hundred degrees Celsius can be applied to the film during microsecond scale rapid photonic curing. It was observed that the peak temperature increased as the thickness of the Ag, the radiant energy density, the radiant power density and the repeat times increased (Fig. 2c).

The highest peak temperature of 867 °C was observed when the radiant energy of 2.7 J/cm^2^ was applied to the multilayer containing a 18 nm Ag layer (Fig. 2d). This high temperature was sufficient to anneal the ITO single-layer, ensure it crystallized^49^, and induce the Ag phase change^50^. Therefore, Xe-FLA has been applied to ITO and amorphous silicon thin films through the digital heat treatment effect on thin films. In addition, for Ag thin film, when thermal annealing is applied, electrical characteristics can be improved by the subsequent diffusion of Ag^32^. This simulation analysis confirmed which conditions were needed to achieve the annealing effect at a sufficient temperature when applying μs-scale rapid photocuring. Since high-temperature annealing occurs in a very short time period during photonic curing, very little thermal energy is transferred to the substrate, minimizing damage^41^.

### Analysis of electrical properties in relation to Xe-FLA conditions

**Figure 3:**
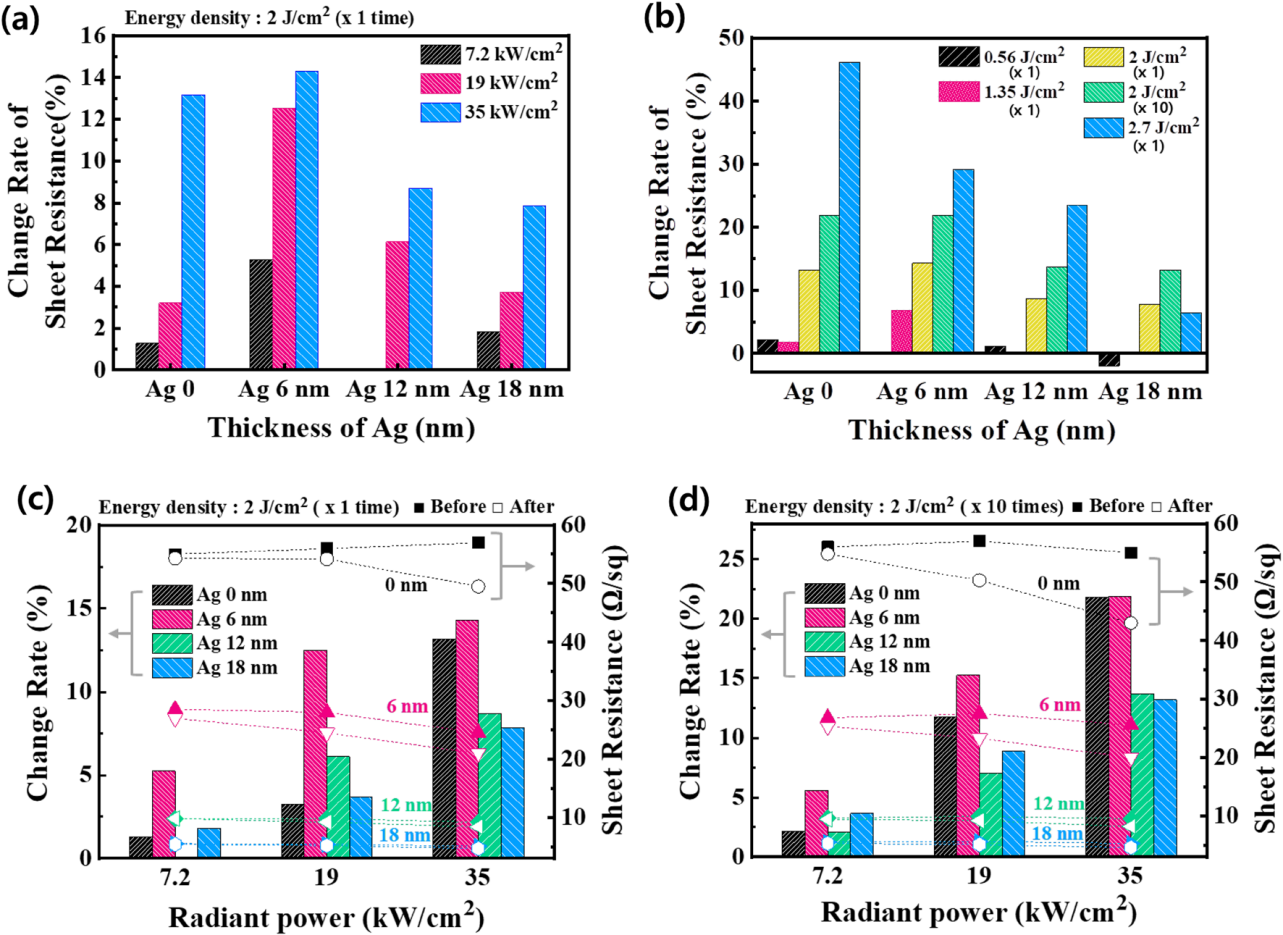
Electrical Properties Analysis according to the Conditions of Xe-FLA: (**a**) Change in sheet resistance for different Ag thicknesses and radiant powers (@ 2 J/cm^2^, × 1 time) (**b**) Change in sheet resistance for different Ag thicknesses and radiant energy densities (0.56 J/cm2–24 kW/cm2, others–35 kW/cm^2^). (**c**) Change in sheet resistance for different Ag thicknesses and radiant powers (@ 2 J/cm^2^, × 1 time). (**d**) Change in sheet resistance for different Ag thicknesses and radiant powers (@ 2 J/cm^2^, × 10times).

Using the conditions determined by the annealing temperature analysis simulation, Xe-FLA-based photonic curing was applied to the ITO–Ag–ITO. Xe-FLA was applied with a radiant energy of 2 J/cm^2^. The rate of change in ITO–Ag–ITO sheet resistance with different Ag thicknesses and radiant power is shown in Fig. 3a and Table S1. The sheet resistance of the OMO structure is composed of the parallel combination of sheet resistance of the ITO and that of Ag. Improving the electrical conductance of the ITO top and bottom oxide layers (Ag thickness of 0 nm) is a crucial factor for improving OMO performance.

As can be seen from the simulation results in Fig. 2, the higher the of Xe-FLA radiation power, the higher the peak temperature of the Ag thin film. As shown in this trend, in Fig. 3a, the sheet resistance improved as the annealing effect increased under the high peak temperature conditions after photonic curing. In addition, in the simulation results, it was confirmed that when Xe-FLA was applied 10 times under each radiant power condition, the reduction in sheet resistance also increased, like the tendency with temperature (Figs. S2, S3). The thinner the Ag layer, the greater the sheet resistance improvement effect, and the effect was maximized at a maximum radiation output of 35 kW/cm^2^.

Figure 3b and Table S2 shows how photonic curing improved sheet resistance based on the thickness of Ag and the radiant energy of Xe-FLA at a radiant power of 35 kW/cm^2^, where the photonic curing effect was the best. The simulation result in Fig. 2d confirms that the peak temperature increased in proportion to the thickness of the Ag and the energy density of the Xe-FLA at a radiant power of 35 kW/cm^2^. The improvement in sheet resistance showed almost the same tendency (Fig. 3b). However, at an Ag thickness of 18 nm, when 2.7 J/cm^2^ (~ 867 °C) of Xe-FLA was applied once, the improvement in sheet resistance was lower than when 2 J/cm^2^ was applied 10 times (~ 762 °C). This result confirms that the photonic curing effect was decreased at a temperature of about 800 °C or higher. In the general thermal annealing method, if the Ag layer is annealed for a long time at a temperature of 300 °C or higher, there is a possibility that the sheet resistance will increase due to aggregation^30^. However, with the photonic curing method in this study, heat was transferred to the electrode for a very short time, in units of μs, and the sheet resistance was improved at a relatively high temperature of about 800 °C.

Figure 3c, d, and Table 1 show the sheet resistance and rate of change according to the radiant power of the Xe-FLA, at a radiant energy of 2 J/cm^2^, which resulted in improvement under all conditions. When the Xe-FLA was irradiated 10 times at a radiant energy of 2 J/cm^2^, the improvement in sheet resistance of the multilayer electrode was the highest, at 21.9% (25.6 → 20.0 ohm/sq) when the Ag thickness was 6 nm. Also, when the Ag thickness was 12 and 18 nm, respectively, similar sheet resistance improvements of 13.7 and 13.2% were observed.

**Table 1:** Improvement in Sheet Resistance after Applying Xe-FLA Photonic Curing.

Energy density (Repeat)	Ag (nm)	Sheet resistance		
		Before Xe-FLA (ohm/sq)	After Xe-FLA (ohm/sq)	Enhancement (%)
2 J/cm^2^ (× 1 time)	0	57.0	49.5	13.2
	6	24.5	21.0	14.3
	12	9.2	8.4	8.7
	18	5.1	4.7	7.8
2 J/cm^2^ (× 10 times)	0	55.0	43.0	21.8
	6	25.6	20.0	21.9
	12	9.5	8.2	13.7
	18	5.3	4.6	13.2

At an Xe-FLA radiant power of 35 kW/cm^2^, which resulted in the best improvement, the improvement in sheet resistance was much greater with a 6 nm layer of Ag, compared to the effect at 12 or 18 nm of Ag. The reason for this is that at Ag 6 nm, Ag is not a continuous film but forms an island-like morphology, and thus has a relatively low sheet resistance. Although it depends on the process conditions, it is generally known that to form a continuous quasi-perfect layer the Ag thickness should be 9 nm or more^51^. Ag thicknesses of 12 and 18 nm produced a quasi-perfect layer, which already had a low sheet resistance before Xe-FLA application, so a relatively low improvement rate was obtained.

To achieve an OMO structure transparent flexible electrode with high transmittance, it is important to form a continuous film while reducing the Ag thickness as much as possible. However, since Ag thin film is generally formed by the Volmer-Weber 3D-island growth model, when Ag is deposited with a thickness of less than 9 nm, a quasi-perfect continuous film cannot be formed, and instead it has a relatively island-like morphology. When Ag is deposited at a thickness of 10 nm or more, a quasi-perfect layer that connects the Ag islands is formed, and the sheet resistance is greatly reduced. However, a thicker Ag layer has a trade-off relationship with decreasing transmittance. Various studies have reported methods of forming a continuous film even with a thin Ag layer, such as inserting a seed layer with high surface energy^52^.

**Figure 4:**
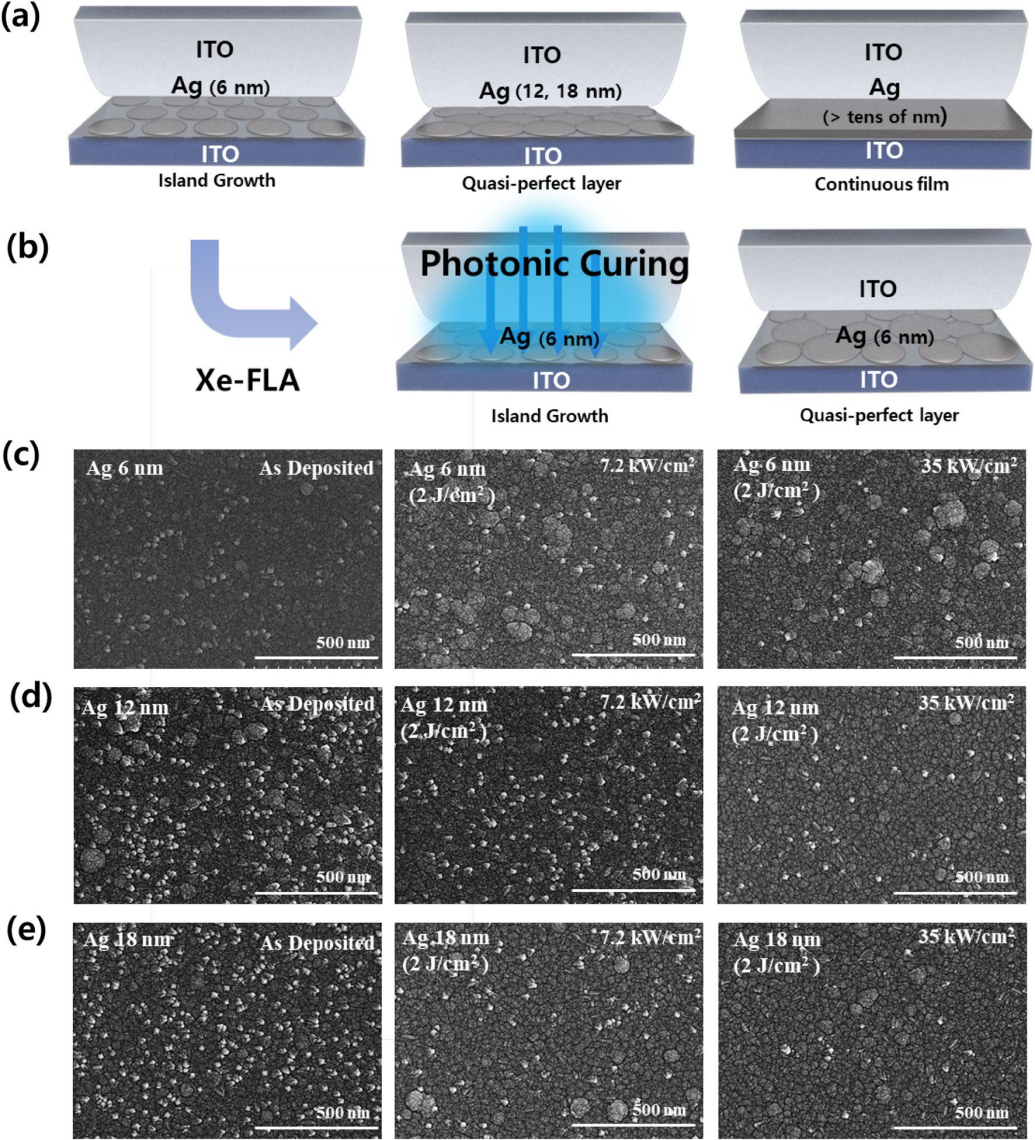
SEM Image of ITO–Ag–ITO Surface Morphology according to Photonic Curing of Xe-FLA: (**a**) Surface morphology for different thicknesses of Ag deposited via the Volmer-Weber 3D-island growth model. (**b**) Quasi-perfect Ag layer formation due to photonic curing effect using Xe-FLA. (**c**) SEM image of different Xe-FLA radiant powers for Ag 6 nm. (**d**) SEM image of different Xe-FLA radiant powers for Ag 12 nm. (**e**) SEM image at different Xe-FLA radiant powers for Ag 18 nm.

In the ITO–Ag–ITO multilayer in this study, 6 nm Ag formed as an island shape, and Ag 12 and 18 nm formed as a quasi-perfect layer (Fig. 4a). According to the SEM results, the surface morphology of ITO-Ag was exactly consistent with that of ITO–Ag–ITO^53^. Therefore, the trends in Ag morphology can be observed using SEM of the ITO–Ag–ITO surface. The sheet resistance significantly decreased at the boundary between Ag thicknesses of 6 and 12 nm (Fig. 3, Table1). Thin Ag grows island-like structures because the surface energy of the substrate is low. It was reported that the morphology and conductivity of an Ag layer sandwiched between ITO were improved due to the diffusion of Ag atoms even after treatment at a high temperature of over 500 °C^32^. In this study, the Xe-FLA diffused the Ag atoms at the same time as the phase change of the Ag, as shown in Fig. 4b, so that a more continuous film was formed.

The SEM image in Fig. 4c confirms that quasi-perfect morphology layers, like those for Ag 12 and 18 nm, were formed even at Ag 6 nm after Xe-FLA was applied (Fig. 4d, e). The SEM image results (Fig. 4c) also show that as the Xe-FLA radiant power increased, a more continuous film was formed, following the trend in sheet resistance reduction. For 12 and 18 nm Ag, although a quasi-perfect layer was initially formed, after the application of Xe-FLA the sheet resistance declined due to the formation of a more perfect film. As the radiant power was increased, SEM images confirmed that the Ag film morphology was more continuous (Fig. 4d, e).

### Analysis of optoelectrical properties according to Xe-FLA conditions

The improvement in the optical properties of the OMO by Xe-FLA photonic curing was analyzed through average transmittance, and the improvement in optoelectrical properties was analyzed using the FOM value. The morphological tendency in the ITO–Ag–ITO after applying Xe-FLA was confirmed by analyzing SEM images. The transmittance of ITO–Ag–ITO is very closely related to the change in Ag morphology. A previously reported, the ITO/Ag/ITO transmittance gradually decreases when Ag is an island-structure, but gradually improves when the Ag forms a quasi-perfect layer.

**Figure 5:**
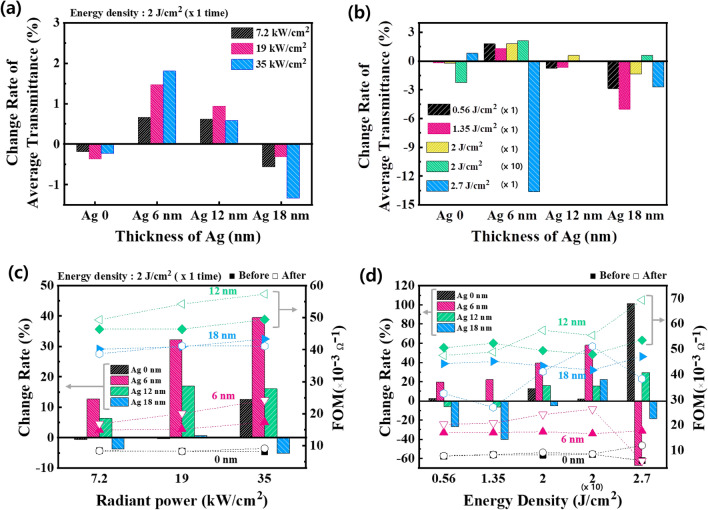
Analysis of optical and FOM properties after Xe-FLA photonic curing: (**a**) Rate of change in average transmittance for different Ag thicknesses and Xe-FLA radiant powers (@ 2 J/cm^2^, × 1 time). (**b**) Rate of change in average transmittance for different Ag thicknesses and Xe-FLA radiant energy densities (0.56 J/cm^2^–24 kW/cm^2^, others–35 kW/cm^2^). (**c**) Rate of change in FOM value with respect to Xe-FLA radiant power and Ag thickness (@ 2 J/cm^2^, × 1 time). (**d**) Rate of change in FOM value with respect to Xe-FLA energy density and Ag thickness (0.56 J/cm^2^–24 kW/cm^2^, others–35 kW/cm^2^).

First, as shown in Fig. 5a, when 2 J/cm^2^ of radiant energy was applied to 6 nm Ag, as the radiant power increased, the average transmittance proportionally improved. This effect followed the same tendency as the previously observed improved resistance. However, for the 12 nm thick Ag, the improvement in transmittance at 19 kW/cm^2^ radiant power was greater than that of 35 kw/cm^2^, and in the case of 18 nm thick Ag, the transmittance decreased under all radiant conditions. When 2 J/cm^2^ of Xe-FLA was applied 10 times, the transmittance also showed additional improvement depending on the conditions, but it was not as significant as the improvement in sheet resistance (Fig. S4).

The transmittance characteristics of the ITO–Ag–ITO were analyzed at different radiant energies, and number of irradiations, at an Xe-FLA radiant power of 35 kw/cm^2^ (Fig. 5b). When the Ag thickness was 6 nm, the transmittance improved up to a radiant energy of 2 J/cm^2^, but the transmittance significantly decreased at 2.7 J/cm^2^. As shown in Fig. S5, under the energy condition of 2.7 J/cm^2^, the transmittance shape of the full spectrum was deformed under all OMO electrode conditions, including the Ag. It can be seen that distortion of the Ag morphology was induced due to the high temperature of the applied 2.7 J/cm^2^ energy. At 2 J/cm^2^, 35 kW/cm^2^ and 10 repeats, the average transmittance increased from 82.1 to 87.3%. On the other hand, for Ag thicknesses of 12 and 18 nm, transmittance decreased under most radiant energy conditions, and improvement in transmittance was only seen under some specific conditions (Ag 12 nm: 2 J/cm^2^ irradiation once, Ag 18 nm: 2 J/cm^2^ irradiation 10 times).

As described above, due to the Xe-FLA photonic curing, at Ag 6 nm, the island-like thin film was rough, and the transmittance improved as a quasi-perfect layer was formed. Therefore, when the Ag was 6 nm, the transmittance improvement also increased according to the power and the number of irradiations at a radiant energy of 2 J/cm^2^ (Fig. S6). On the other hand, since the 12 nm and 18 nm Ag formed a quasi-perfect layer from the beginning, the Xe-FLA did not significantly improve transmittance, but when the roughness was improved depending on the conditions of irradiation, improved transmittance was observed (Fig. S6).

The value of FOM for both the conductivity and transmittance of the transparent electrode is important. Figure 5c and Table 2 show the FOM improvement ratio and values according to radiant power when Xe-FLA photonic curing of 2 J/cm^2^ was applied. The 6 nm-thick Ag, which had the greatest electro-optical improvement after photonic curing with Xe-FLA, showed the greatest FOM improvement, and the 12 nm-thick Ag showed the second highest FOM improvement. However, 18 nm thick Ag showed a somewhat decreased FOM due to the decrease in transmittance, despite the improved conductance. When the radiant energy of 2 J/cm^2^ was applied 10 times, the FOM improvement differed depending on the conditions, and in particular, the 6 nm Ag showed a maximum FOM value improvement of 58% under a radiant power of 35 kW/cm^2^ (Fig. S7, Table 2).

**Table 2:** Improvement in FOM Value after Applying Xe-FLA photonic curing.

Energy density (Repeat)	Ag (nm)	FOM		
		Before Xe-FLA (ohm/sq)	After Xe-FLA (ohm/sq)	Enhancement (%)
2 J/cm^2^ (× 1 time)	0	8.12	9.14	12.61
	6	17.34	24.19	39.54
	12	49.41	57.37	16.10
	18	43.32	41.10	− 5.12
2 J/cm^2^ (× 10 times)	0	8.42	8.59	2.10
	6	16.59	26.22	58.00
	12	47.85	55.39	15.74
	18	41.69	51.02	22.38

The effect of photonic curing at a radiant energy at 35 kW/cm^2^, which had the best FOM improvement, is shown in Fig. 5d. At a Ag thickness of 6 nm, the FOM improvement was proportional to the energy of 2 J/cm^2^. However, at an energy of 2.7 J/cm^2^, the FOM value decreased because the transmittance decreased after Xe-FLA. Because of the excellent basic electrical characteristics of the multilayer with an Ag layer thickness of 12 nm, the maximum value of FOM was 69.33 under photonic curing conditions of 2.7 J/cm^2^ with a radiant power of 35 kW/cm^2^.

**Figure 6:**
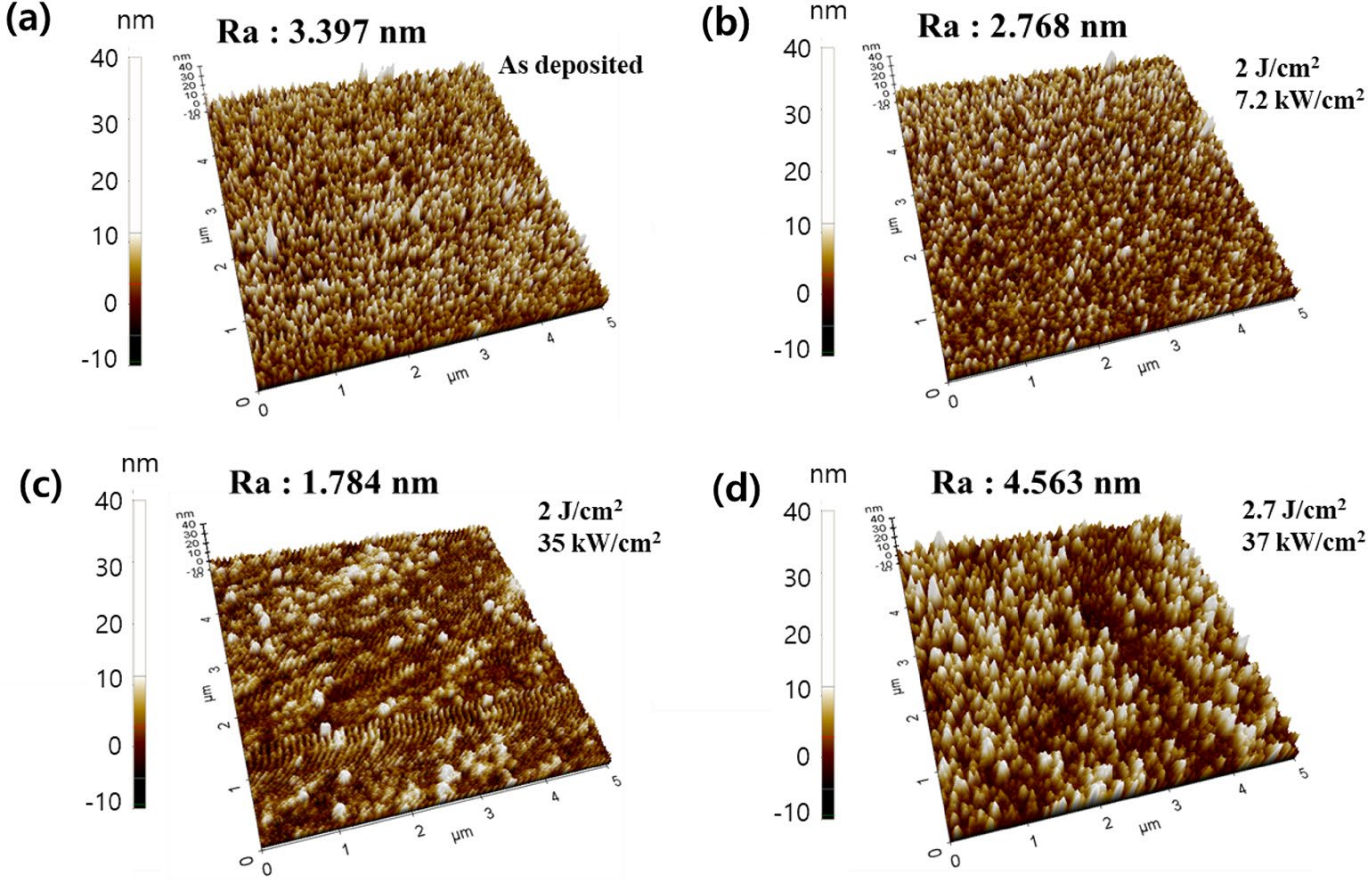
AFM images of ITO (48 nm)-Ag (12 nm)-ITO (48 nm) multilayer, (**a**) as deposited (Ra = 3.397 nm), (**b**) 2 J/cm^2^, 7.2 kW/cm^2^, 1 repeat (Ra = 2.768 nm), (**c**) 2 J/cm^2^, 35 kW/cm^2^, 1 repeat (Ra = 1.784 nm), and (**d**) 2.7 J/cm^2^, 37 kW/cm^2^, 1 repeat (Ra = 4.563 nm).

To confirm the effect of Xe-FLA on the surface of the multilayer, an atomic force microscope (AFM) was used to observe the multilayer morphology. The multilayer with an Ag thickness of 12 nm, which had the maximum FOM value, was selected for AFM observation (Fig. 6). The AFM image confirmed that the surface roughness was improved after photonic curing at 2 J/cm^2^ compared to before Xe-FLA annealing. The photonic curing results confirmed that the conditions for improving transmittance and the conditions for improving surface roughness were the same (Figs. 5b, 6). However, after photonic curing at 2.7 J/cm^2^, the transmittance showed similar or decreased results, because the surface roughness deteriorated slightly (Figs. 5b, 6d). These results indicate that Xe-FLA photonic curing can improve the surface roughness of Ag depending on the conditions, and as a result, the FOM value increased as the transmittance of the multilayer improved.

In this study it was possible to obtain a transparent electrode with a high FOM value by inducing rapid photonic curing effect at high temperature, in the μs time scale, using Xe-FLA, This opens the future possibility of a high-throughput roll-to-roll process, because an extremely low thermal budget was employed. Compared to the transparent ITO–Ag–ITO flexible electrode studied with the previous annealing method, a high FOM value was achieved, even with a very short photonic curing time, as shown in Table 3.

**Table 3:** Comparison of annealing conditions and key parameters of ITO–Ag–ITO electrodes.

Structure	FOM (10^−3^* ohm^−1^)	Resistance (ohm/sq)	Transmittance (%)	Temperature (℃)	Time (sec.)	Thermal budget (Temp. * Time)	Refs.
ITO/Ag/ITO	69.3	6.5	92.3	727	0.0001	0.0727	This work
ITO/Ag/ITO	85.0	6.0	90.0	350	900	315,000	^29^
ITO/Ag/ITO	66.0	10.0	96.0	500	300	150,000	^30^
ITO/Ag/ITO	64.6	6.4	91.4	300	3600	1,080,000	^31^
ITO/Ag/ITO	121.0	3.0	90.2	600	1800	1,080,000	^32^
ITO/Ag/ITO	106.0	3.8	91.3	600	300	180,000	^33^
ITO/Al–Ag/ITO	76.4	2.9	86.1	400	300	120,000	^34^
ITO/Ag/ITO	46.4	6.2	88.2	300	300	90,000	^35^

## Conclusion

In summary, we demonstrated a high-quality ITO–Ag–ITO transparent electrode that can be fabricated with high-throughput using Xe-FLA rapid photonic curing, with an extremely low thermal budget. The Xe-FLA annealing temperature was proportional to radiant power, radiant energy, number of irradiations, and the thickness of the Ag, and the thermal budget condition allowed annealing in the range of 100–900 °C for a very short time. When the photonic curing conditions were applied to multilayer ITO–Ag–ITO, the sheet resistance was greatly improved in proportion to the annealing temperature up to about 800 °C. For Ag thicknesses of 6 and 12 nm in the OMO transparent electrode, the highest sheet resistance improvement was observed at annealing conditions (35 kW/cm^2^, 2.7 J/cm^2^, × 1 time) of about 700 °C.

Since the Ag layer is grown by the Volmer-Weber 3D-island growth model, the Ag layer is not continuously connected at thicknesses below 10 nm, resulting in high sheet resistance. However, after Xe-FLA, SEM images confirmed that a quasi-perfect layer had been formed. Because the Ag thin films were continuously connected, the sheet resistance was greatly improved. In addition, Xe-FLA improved the surface roughness of the ITO–Ag–ITO, and transmittance was also improved. When Xe-FLA was applied, the FOM value of the multilayer electrode with an Ag thickness of 6 nm increased by 58%, and when the Ag thickness was 12 nm, the FOM value reached the maximum value of 69.3.

## Materials and methods

### ITO–Ag–ITO fabrication

In this experiment, the in-line magnetron sputtering method was used to prepare the OMO multilayer, because magnetron sputtering can deposit thin films uniformly over a large area at a low temperature^51,54^. The OMO multilayer was deposited on a glass substrate (75 × 16 × 1 mm^3^) by in-line magnetron sputtering. Prior to deposition, the substrate was cleaned with acetone in an ultrasonic cleaner, and then washed with DI water. ITO and Ag layers were deposited using a 540 × 165 mm^2^ ITO target (99.99%) and 4-inch Ag target (99.99%), respectively. The deposition conditions of the ITO and Ag layers are shown in Tables S3 and S4, respectively.

In order to obtain an ITO with high conductivity and stable optoelectrical characteristics, and to prevent excessive oxygen vacancies from being generated, oxygen was appropriately added during the deposition process^55,56^. In order to ensure the uniformity of the deposition, a scanning method was used in the sputtering process. This means that the substrate is moved through the target during the deposition process. The scanning time was set as a variable in the Ag deposition, in order to observe the influence of different thicknesses of Ag layer after annealing the ITO–Ag–ITO multilayer. A high vacuum condition was ensured before sputtering, and the process was carried out at air condition.

### Characterization of the OMO electrode

Changes in sheet resistance, transmittance and surface morphology according to the annealing process were observed by 4-point probe, ultraviolet–visible spectroscope (UV–vis) and atomic force microscope (AFM), respectively.

### Xe-Flash lamp annealing

PulseForge 1300 (PulseForge Corporation) was used for the flash lamp annealing treatment. As shown in Table S5, four different radiant energies, 0.56, 1.35, 2 and 2.7 J/cm^2^, were used in the flash lamp annealing experiment, corresponding to different radiant power and pulse envelopes. Three different radiant powers, 7.2, 19 and 35 kW/cm^2^, were adopted under the 2 J/cm^2^ condition.

## Supplementary Information

Supplementary Information.

## Data Availability

The datasets used and/or analyzed during the current study available from the corresponding author on reasonable request.
