# Supplementary material for: Rapid photonic curing effects of xenon flash lamp on ITO–Ag–ITO multilayer electrodes for high throughput transparent electronics
Source: Sci Rep. 2023 Jan 19;13:1042. doi: 10.1038/s41598-023-27942-4 (PMC9852449; doi:10.1038/s41598-023-27942-4)
Supplement: Supplementary file 1 — Supplementary Information. [file 41598_2023_27942_MOESM1_ESM.docx]

**Rapid Photonic Curing Effects of Xenon Flash Lamp on ITO-Ag-ITO Multilayer Electrodes for High throughput Transparent Electronics**

**Zhenqian Zhao^1^,** **Alex Rose^3^, Sang Jik Kwon^1^, Yongmin Jeon^2*^ and Eou-Sik Cho^1^****^*^**

^1^Department of Electronics Engineering, Gachon University, Seongnam 13120, Republic of Korea.

^2^Department of Biomedical Engineering, Gachon University, Seongnam 13120, Republic of Korea.

^3^PulseForge Corporation, Seoul 04070, Republic of Korea

^*^ Corresponding author: [yongmin@gachon.ac.kr](mailto:yongmin@gachon.ac.kr) (Yongmin Jeon),

[es.cho@gachon.ac.kr](mailto:es.cho@gachon.ac.kr) (Eou-Sik Cho)

**Supplementary Figures**

**Fig. S1. Average transmittance of visible light of multilayer according to Ag thickness.**


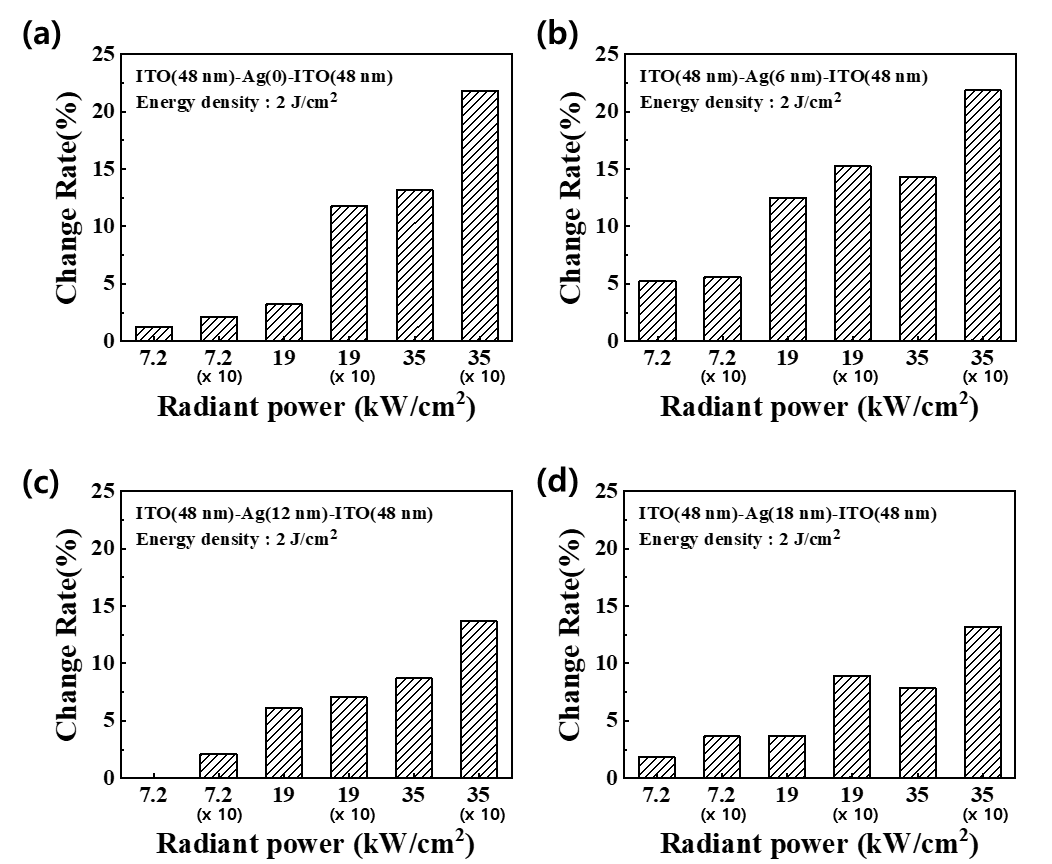


**Fig. S2. Reduction in sheet resistance according to radiant power and number of irradiations.**

**Fig. S3. Rate of change in sheet resistance when Xe-FLA with a radiant energy of 2J/cm^2^ was irradiated 10 times**

**Fig. S4. Average rate of change in transmittance when Xe-FLA with a radiant energy of 2J/cm^2^ was irradiated 10 times**


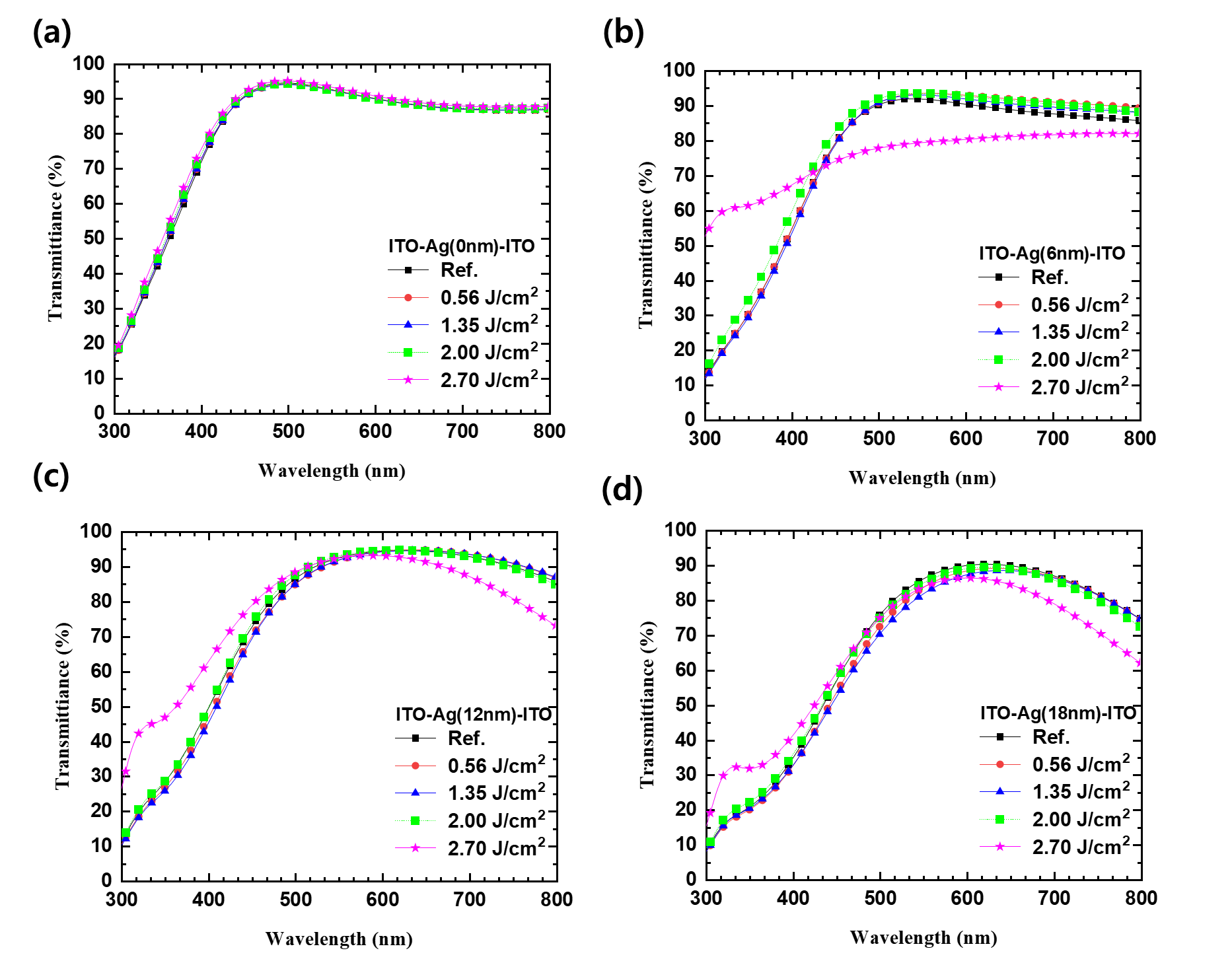


**Fig. S5. Full spectrum of transmittance of ITO-Ag-ITO after application of Xe-FLA for each condition**


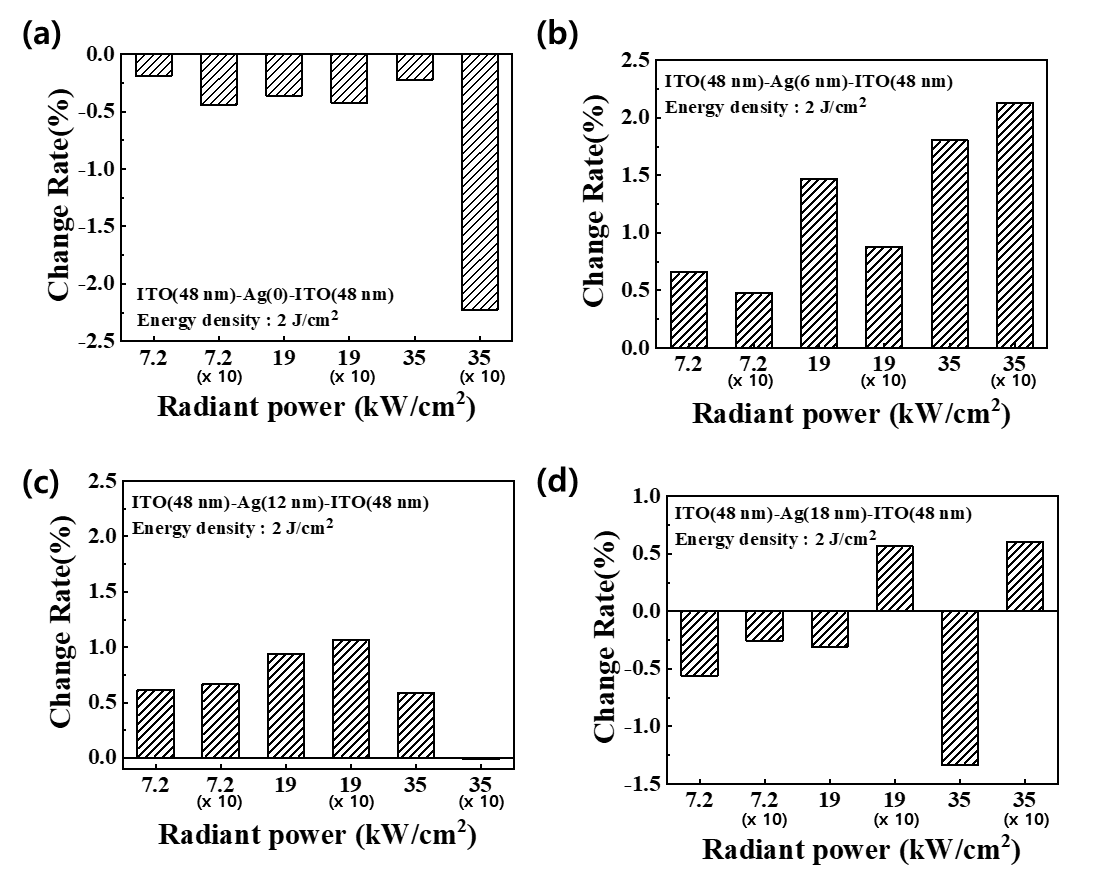


**Fig. S6. Rate of change in transmittance according to Xe-FLA radiant power and number of irradiations.**

**Fig. S7. FOM improvement when Xe-FLA with a radiant energy of 2J/cm2 was applied 10 times**

**Supplementary Tables**

**Table S1.** **Resistance characteristics by power density and Ag thickness of Xe-FLA**

| **Power Density**  **(kW/cm^2^ @2J/cm^2^)** | **Ag Thick. (nm)** | **Resistance (ohm/sq)** | | |
| --- | --- | --- | --- | --- |
|  |  | **Before** | **After** | **%** |
| **7.2 (x 1 time)** | 0.0 | 55.0 | 54.3 | 1.27 |
|  | 6.0 | 28.5 | 27.0 | 5.26 |
|  | 12.0 | 9.8 | 9.8 | 0.00 |
|  | 18.0 | 5.5 | 5.4 | 1.82 |
| **7.2 (x 10 time)** | 0.0 | 56.0 | 54.8 | 2.14 |
|  | 6.0 | 26.8 | 25.3 | 5.60 |
|  | 12.0 | 9.6 | 9.4 | 2.08 |
|  | 18.0 | 5.5 | 5.3 | 3.64 |
| **19 (x 1 time)** | 0.0 | 56.0 | 54.2 | 3.21 |
|  | 6.0 | 28.0 | 24.5 | 12.50 |
|  | 12.0 | 9.8 | 9.2 | 6.12 |
|  | 18.0 | 5.4 | 5.2 | 3.70 |
| **19 (x 10 time)** | 0.0 | 57.0 | 50.3 | 11.75 |
|  | 6.0 | 27.5 | 23.3 | 15.27 |
|  | 12.0 | 9.9 | 9.2 | 7.07 |
|  | 18.0 | 5.6 | 5.1 | 8.93 |
| **35 (x 1 time)** | 0.0 | 57.0 | 49.5 | 13.16 |
|  | 6.0 | 24.5 | 21.0 | 14.29 |
|  | 12.0 | 9.2 | 8.4 | 8.70 |
|  | 18.0 | 5.1 | 4.7 | 7.84 |
| **35 (x 10 time)** | 0.0 | 55.0 | 43.0 | 21.82 |
|  | 6.0 | 25.6 | 20.0 | 21.88 |
|  | 12.0 | 9.5 | 8.2 | 13.68 |
|  | 18.0 | 5.3 | 4.6 | 13.21 |

**Table S2.** **Resistance characteristics by energy density and Ag thickness of Xe-FLA**

| **Energy Density**  **(J/cm^2^)** | **Ag Thick. (nm)** | **Resistance (ohm/sq)** | | |
| --- | --- | --- | --- | --- |
|  |  | **Before** | **After** | **%** |
| **0.56** | 0.0 | 60.3 | 59.0 | 2.16 |
|  | 6.0 | 25.0 | 25.0 | 0.00 |
|  | 12.0 | 9.0 | 8.9 | 1.11 |
|  | 18.0 | 5.0 | 5.1 | -2.00 |
| **1.35** | 0.0 | 56.0 | 55.0 | 1.79 |
|  | 6.0 | 25.0 | 23.3 | 6.80 |
|  | 12.0 | 8.7 | 8.7 | 0.00 |
|  | 18.0 | 4.9 | 4.9 | 0.00 |
| **2.00 (x 1 time)** | 0.0 | 57.0 | 49.5 | 13.16 |
|  | 6.0 | 24.5 | 21.0 | 14.29 |
|  | 12.0 | 9.2 | 8.4 | 8.70 |
|  | 18.0 | 5.1 | 4.7 | 7.84 |
| **2.00 (x 10 times)** | 0.0 | 55.0 | 43.0 | 21.82 |
|  | 6.0 | 25.6 | 20.0 | 21.88 |
|  | 12.0 | 9.5 | 8.2 | 13.68 |
|  | 18.0 | 5.3 | 4.6 | 13.21 |
| **2.70** | 0.0 | 78.0 | 42.0 | 46.15 |
|  | 6.0 | 24.0 | 17.0 | 29.17 |
|  | 12.0 | 8.5 | 6.5 | 23.53 |
|  | 18.0 | 4.7 | 4.4 | 6.38 |

**Table S3.** **Sputtering deposition conditions of ITO layers.**

| ITO deposition condition | Values |
| --- | --- |
| Power / Power density | DC 2 kW / 2.24 W/cm^2^ |
| Ar gas | 50 sccm |
| O_2_ gas | 1.2 sccm (O_2_ ratio : 2.34%) |
| Pressure | 4 mTorr |
| Scanning speed | 60 cm/min |
| Scanning time | 1 scanning |
| Base chamber vacuum | 8.0 × 10^-6^ Torr |
| Temperature | Room temperature |

**Table S4. Sputtering deposition conditions of Ag layers.**

| Ag deposition condition | Values |
| --- | --- |
| Power / Power density | RF 60 W / 0.74 W/cm^2^ |
| Ar gas | 30 sccm |
| Pressure | 3 mTorr |
| Scanning speed | 150 cm/min |
| Scanning time | 0, 1, 2, 3 scanning |
| Base chamber vacuum | 8.0 × 10^-6^ Torr |
| Temperature | Room temperature |

**Table S5. Flash lamp annealing conditions on ITO-Ag-ITO multilayer.**

| Radiant energy [J/cm^2^] | Radiant power [kW/cm^2^] | Pulse envelop [μs] | Repeat  [count] | Fire rate [Hz] |
| --- | --- | --- | --- | --- |
| 0.56 | 24 | 40 | 1 | 1 |
| 1.35 | 37 | 65 | 1 | 1 |
| 2 | 7.2 | 283 | 1 | 1 |
|  |  |  | 10 | 2 |
|  | 19 | 133 | 1 | 1 |
|  |  |  | 10 | 2 |
|  | 35 | 84 | 1 | 1 |
|  |  |  | 10 | 2 |
| 2.7 | 37 | 100 | 1 | 1 |
